# Gbm.auto: A software tool to simplify spatial modelling and Marine Protected Area planning

**DOI:** 10.1371/journal.pone.0188955

**Published:** 2017-12-07

**Authors:** Simon Dedman, Rick Officer, Maurice Clarke, David G. Reid, Deirdre Brophy

**Affiliations:** 1 Marine and Freshwater Research Centre, Galway-Mayo Institute of Technology, Galway, Ireland; 2 Marine Institute, Rinville, Oranmore, Co. Galway, Ireland; Northwest Fisheries Science Center, UNITED STATES

## Abstract

**Boosted Regression Trees. Excellent for data-poor spatial management but hard to use:**

Marine resource managers and scientists often advocate spatial approaches to manage data-poor species. Existing spatial prediction and management techniques are either insufficiently robust, struggle with sparse input data, or make suboptimal use of multiple explanatory variables. Boosted Regression Trees feature excellent performance and are well suited to modelling the distribution of data-limited species, but are extremely complicated and time-consuming to learn and use, hindering access for a wide potential user base and therefore limiting uptake and usage.

**BRTs automated and simplified for accessible general use with rich feature set:**

We have built a software suite in R which integrates pre-existing functions with new tailor-made functions to automate the processing and predictive mapping of species abundance data: by automating and greatly simplifying Boosted Regression Tree spatial modelling, the *gbm*.*auto* R package suite makes this powerful statistical modelling technique more accessible to potential users in the ecological and modelling communities. The package and its documentation allow the user to generate maps of predicted abundance, visualise the representativeness of those abundance maps and to plot the relative influence of explanatory variables and their relationship to the response variables. Databases of the processed model objects and a report explaining all the steps taken within the model are also generated. The package includes a previously unavailable Decision Support Tool which combines estimated *escapement biomass* (the percentage of an exploited population which must be retained each year to conserve it) with the predicted abundance maps to generate maps showing the location and size of habitat that should be protected to conserve the target stocks (candidate MPAs), based on stakeholder priorities, such as the minimisation of fishing effort displacement.

**Gbm.auto for management in various settings:**

By bridging the gap between advanced statistical methods for species distribution modelling and conservation science, management and policy, these tools can allow improved spatial abundance predictions, and therefore better management, decision-making, and conservation. Although this package was built to support spatial management of a data-limited marine elasmobranch fishery, it should be equally applicable to spatial abundance modelling, area protection, and stakeholder engagement in various scenarios.

## Introduction

### Spatial management of data-limited species

Some of the key barriers to implementation of scientific research are accessibility of evidence, quality of evidence, and organisational capacity/resources [1]. A lack of data commonly complicates conservation of marine and terrestrial species [2]. Marine spatial management typically involves the selection of appropriate Marine Protected Areas (MPAs) [3]. Fisheries managers and scientists recommend various spatial management tools to support MPA selection [4–6]. These methods generally involve predictive mapping of species distribution and abundance in relation to available habitat and human activities such as fishing (e.g. [7–10]), and there are a number of such approaches available. However, predicting abundance (especially to fine spatial scales) is often hampered by lack of data [8,11]. Evaluating the suitability of an MPA is often difficult due to incomplete knowledge of specific ecologically important habitats such as nursery and spawning areas [12,13], and uncertainty regarding species movement and larval dispersal patterns [14,15]. MPAs that ignore the biology of the species to be conserved (e.g. home range size) may be inappropriately sized and subsequently fail [16,17].

### BRTs compared to other spatial management tools

Various spatial management tools exist, with different capabilities and strengths, one of which is Boosted Regression Tree (BRT) modelling. Compared to other such tools, BRTs are robust to poor or absent data, which Marxan may not be [18–20], and can use abundance data, unlike Maximum Entropy (MaxEnt) models. They are relatively insensitive to the effects of missing predictor values, outliers, and multicollinearity [21,22] and can accommodate large numbers of explanatory variables without penalty. They can provide more robust predictions than generalised linear and additive models (GLMs and GAMs)[20], with less variance (oversensitivity to noise leading to overfitting/imprecision) and bias (false assumptions in the algorithm leading to underfitting/inaccuracy), a lower risk of misspecification, and the ability to model complex interactions (see comparative evaluation in [23]). BRTs have a demonstrated ability to generate predicted abundance maps at fine spatial scales for data-poor stocks [7,24,25], as well as for age- and gender-subsets of those stocks [26]. Species may be managed as an assemblage [27,28], which BRTs can assist with [7,29].

### The case for simplified BRTs

Functions exist in the repository of the R programming language [30] to run BRTs on sparse datasets [31,32], and one can then display the resulting predicted abundance data tables using either a Geographic Information System (GIS) package or functions within R (e.g. *mapplots*), thus producing fine-spatial-scale predicted abundance maps for data-poor stocks and their subsets. However, despite guidance existing for the stepwise running of some of these functions [33], much time and technical proficiency is required to understand and run the complicated programming functions in a piecemeal fashion. Even when successfully run, BRTs are complex, acknowledged as being “challenging to understand”, with the model object often treated as a “black box” [31]. The application of these methods therefore requires time, technical and financial resources that are often unavailable to scientists or marine managers (e.g. [1,34,35]), and could disincentivise potential users. A software tool that automates and simplifies the BRT mapping process could greatly reduce the barriers preventing access to these methods by the potential user base, and thus increase the uptake of this high performance method [20], assisting and improving fisheries management.

### Managing multiple species or life history groups

Protection of spawning and/or nursery grounds is often proposed as a spatial management solution for species conservation [4,12,36,37].This can be achieved by mapping the abundance of a particular life history stage (such as mature females (e.g. [38]) and BRT mapping can support this approach. However, simply summing predicted abundance maps (e.g. [7]), sees more abundant (probably less threatened) subsets obfuscating less abundant (more threatened) subsets. There is a need for a software function that allows users to generate synthesis maps for multiple species or life history groups and to weight each subset according to conservation priorities, to facilitate management of multiple species and their subsets.

### Decision support tools for MPA selection

Generating predictive maps of species distribution and abundance from the available data only addresses half of the problem of species conservation within an impacted multi-use environment. While MPAs can improve decision making [39], a common mistake made when designing them is failing to acknowledge that conservation plans are prioritisations [40]. This means that socioeconomic costs must be considered [14,41], not only to the primary stressor (e.g. fishermen) but all affected parties (e.g. tourism, oil and gas extraction, etc.). Insufficient stakeholder engagement—often by involving them only at the final stage of the process—is a common reason for MPAs to fail [16,39,42]. Unfortunately, the tools and guidance required to incorporate biologically-derived MPA candidates into a multi-stakeholder environment are lacking ([43,44] in [45]). While tools to support qualitative evaluation of candidate MPAs are available [35,45], they could be enhanced by the incorporation of quantitative metrics (e.g. Maximum Sustainable Yield (MSY), fishing effort) [43,46]. There is a need for a DST that can generate MPA candidates across a whole region, which weigh harvest-limit-based conservation against quantified displacement of the stressor, e.g. fishing effort, catch per unit effort (CPUE), or profit. This would allow much-needed [43] evaluation of trade-offs within a framework of scientist, manager, and stakeholder discussion. A review of 39 MPA-generation and decision support tools found that most were only usable by scientists, and custom-tailored rather than generic; they concluded a practical and simple tool is required [41].

### Aims

In this paper we present the *gbm*.*auto* package that we have written, and describe its usage and functionality. Its core function—also named *gbm*.*auto*—assists decision makers in mapping species distributions by addressing the need for a simplified BRT mapping process. It reduces thousands of lines of code to as few as two, and automatically outputs maps of predicted abundance and representativeness maps of those abundance maps. It produces bar plots of the relative influence of explanatory variables, dot and line plots describing the relationships between explanatory variables and response variables, and databases of the processed model objects. These outputs provide insight into the factors underlying observed distribution and abundance patterns that can be disseminated to all stakeholders to catalyse and enrich discussions. Finally, the function produces a report detailing the steps taken by the model, the optimal BRT argument combinations, the size of variable influence and variable interactions, and performance statistics of the final models. This provides the user with the important technical information the model used, and allows them to evaluate the robustness of the outputs

In running the *gbm*.*auto* function, functions *gbm*.*rsb*, *gbm*.*map*, and *gbm*.*basemap* are called and run. Users can test optimal parameters beforehand with *gbm*.*bfcheck*, and run any of the aforementioned functions independently. *Gbm*.*cons* can then be used to generate maps synthesising the predicted abundances of multiple subset components of stocks, such as juveniles and mature females, a highly desirable output for marine managers.

Separate from the machine-learning spatial-modelling function *gbm*.*auto*, the other major function within the *gbm*.*auto* package is a DST called *gbm*.*valuemap*. The function combines an MSY-based conservation value metric with the predicted abundance maps from *gbm*.*auto* to propose MPAs that ensure the target stocks are harvested sustainably, while also minimising fishing effort displacement as a proxy for stakeholder priorities. This bridges the gap between species distribution modelling and decision-making, whereas most existing tools specialize in one or the other.

This is a DST software package that vastly simplifies the process of generating fine-spatial-scale predicted abundance maps for data-poor species, then produces MPAs combining fisheries stock science with quantified stakeholder preferences. The process of spatial conservation is thus facilitated with a rich collection of tools and outputs. Scientists, conservation managers and policymakers can benefit from the power of these statistical methods without a significant upfront penalty of time and effort. The availability of this specialist software package will increase the uptake of this class-leading analysis method, vastly the diminishing the work required to produce a suite of valuable outputs, and facilitating the engagement of stakeholders into the management process.

## Guide to software functions

We wrote the gbm.auto package in R. It incorporates existing packages calibration, roc, gbm.predict.grids (from [31]’s appendix, bundled into gbm.utils by Dedman), beepr, dismo, gbm, mapplots, mgcv, raster, rgdal and vegan, and functions also built by the authors for this task: gbm.map, gbm.basemap, gbm.rsb, gbm.cons, gbm.valuemap, gbm.bfcheck and gbm.loop. All R source code is publicly available via GitHub (see ‘Availability’ and S3 File).

**Fig 1:**
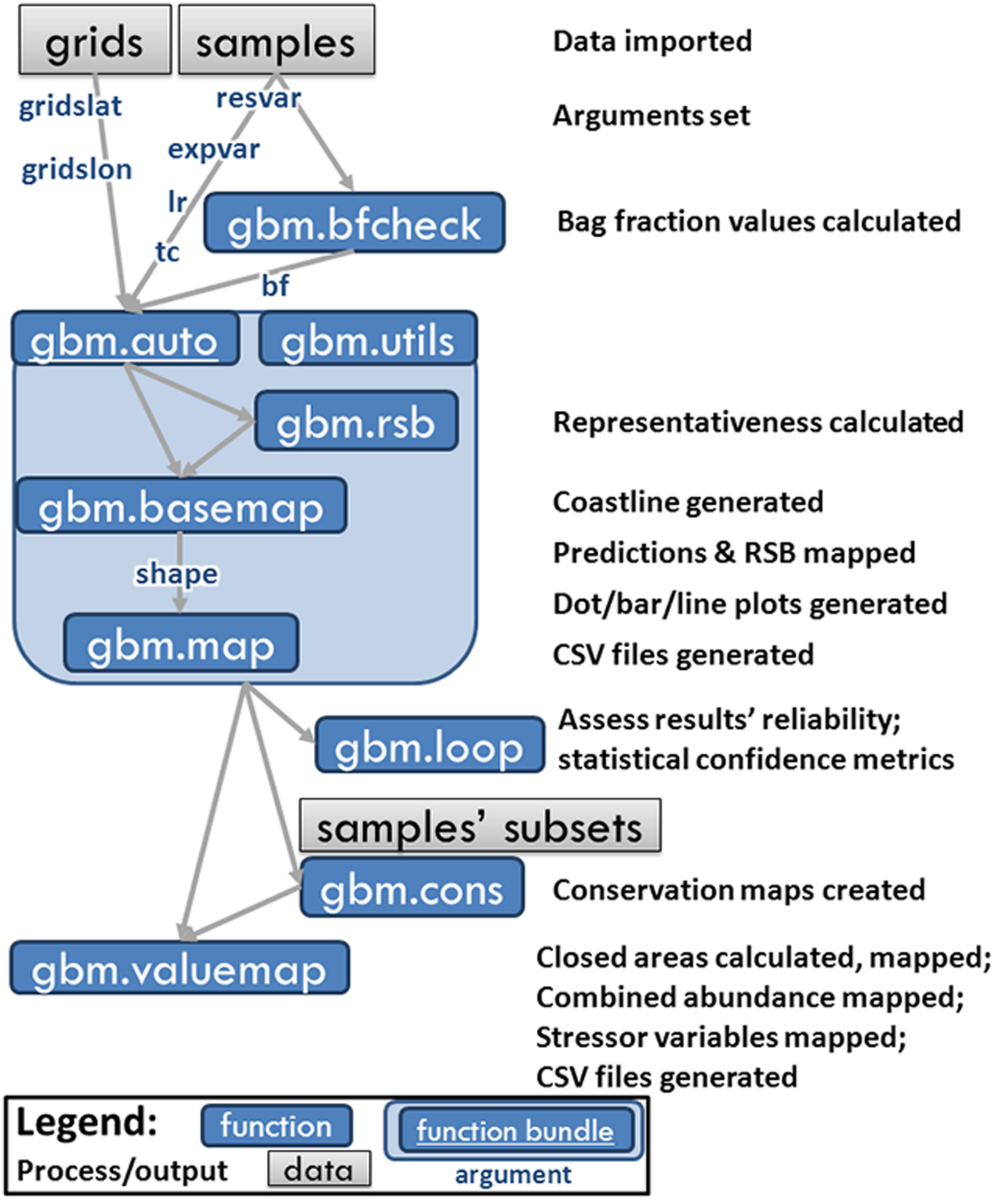
Conceptual diagram of main modelling processes and outputs.

Below follows a brief explanation of the usage of BRTs which are the central engine of the *gbm*.*auto* modelling process, then an introduction to the case study for which this software was initially developed, and a description of the required data format. Subsequently, the individual functions are introduced and explained. See Fig 1 for a schematic describing the functionality of gbm.auto functionality.

### Delta log-normal BRT models for abundance predictions

A high proportion of zeros and few very high values are common features of marine species abundance data acquired through fisheries sampling methods such as trawling; in fisheries science these issues are commonly addressed using a delta log-normal approach. Delta log-normal boosted regression trees split the data into zero/non-zero catches (a binary response variable), and log-normalised non-zero catches (a continuous variable representing abundance). The model then uses machine learning to infer a relationship between the explanatory variables and these two sets of response variables, separately predicting the probability of occurrence, and the expected abundance (this is the delta process, also known as a hurdle model). The abundance variable is reverse log-transformed and the two datasets combined into one abundance probability metric which is mapped to the whole study area, including areas with explanatory data but no response data. For more detailed explanation see Reference 7] and references therein, including R packages; the conceptual diagram (Fig 1); and [26,29] for expounded applications to these sample data. An explanation of the history and mathematics of BRTs is comprehensively covered by Reference 31]. If data are not zero-inflated and long-tailed, the log-normalisation and reversal stages can be omitted, and the BRT run only a single time using the distribution model most appropriate for the data (e.g. binomial, Gaussian, Poisson), as opposed to using the delta/hurdle model. Users are able to specify which model(s) *gbm*.*auto* should direct BRTs to fit to their data, if they wish.

### Sample dataset

Management bodies and scientists recommend exploration of novel spatial approaches to conserve vulnerable elasmobranch species, as an alternative to typical Total Allowable Catch (TAC) based management, which often fails such species [4,12]. Of the 1088 chondrichthyan species in the IUCN Red List, 480 (44%) are categorised as Data Deficient [47] and a high percentage of these could qualify as threatened [48]. Chondrichthyans are thus appropriate candidates for this data-poor spatial approach [49,50].

In the worked example we use a dataset of CPUEs from surveys for four rays (cuckoo ray (*Leucoraja naevus*), thornback ray (*Raja clavata*), blonde ray (*Raja brachyura*) and spotted ray (*Raja montagui*)) from 1447 survey stations in the Irish Sea over 12 years. Relationships with fishing pressure, environmental correlates (bottom temperature, depth, salinity, current speed, substrate grain size, and distance from shore), and juvenile ray and eggcase reducing variables (fishing effort, predatory fish CPUE, scallop dredging effort, whelk CPUE) are used to map predicted CPUEs for the whole study area. The example data set and images can be retrieved from GitHub (see ‘Availability’ section).

While the example pertains to marine fishes, the *gbm*.*auto* approach may be equally applicable for the management of marine, estuarine, riverine, or terrestrial species.

### Input data format

For predicting abundance, two data tables are expected–‘samples’, containing the response variable (e.g. CPUE of fish) and predictor variables (e.g. environmental variables such as temperature, depth, etc.) at various sites; and ‘grids’, containing the same environmental variables at (ideally regularly-spaced (gridded)) sites where the response variable was not measured. ‘grids’ can be omitted if the objective is not to predict abundance at new sites. A model object of the relationship between predictor and response variables will still be created, and the dot, bar, and line plots, and progress report will all be generated in R. In our example, these data come from a variety of sources; an explanation of how they were compiled and treated is provided in ‘Data sources and processing’ (S2 File).

### Worked examplePre-run parameter scoping with *gbm*.*bfcheck*

Before starting often-lengthy *gbm*.*auto* runs, the *gbm*.*bfcheck* function allows users to calculate the minimum binary and Gaussian BRT bag fraction sizes (the portion of the data that is randomly selected at each iteration to train the model), as insufficiently sized datasets or over-large subsampling rates can cause BRT runs to fail. Its arguments are ‘samples’, ‘resvar’ and ‘ZI’, all as per *gbm*.*auto*.

gbm.bfcheck(samples = mysamples, resvar = 11) # Run code with defaults

### Abundance predictions with *gbm*.*auto*

The *gbm*.*auto* code loops through the arguments resvar, tc, lr and bf, either left as default or provided by the user, then checks whether the response variable data are zero inflated, and that the data are correctly formatted. For a binomial distribution or delta log-normal approach (which includes binomial), zeroes are expected to be present in the raw data, denoting zero catches. Column names in the samples data must match those in the grids data. Binary and non-zero Gaussian data vectors are then created from the original samples data, for input to the subsequent BRT model runs.

The remaining argument loops are then begun, with bag fraction nested within learning rate nested within tree complexity. Binomial and Gaussian BRTs are run on the binary and non-zero Gaussian data respectively (assuming the model families have been left at their defaults), with the best-performing combination of arguments selected then tested for simplicity, in case it performs better with any explanatory variables omitted.

Line plots of partial deviance are created, first all on one matrix figure, then for each variable separately, for binary and Gaussian as usual. Next are dot plots of the spread of partial deviance against the explanatory variable values. The influence of each variable’s contribution to the model is then tabulated and saved as a comma-separated-values (csv) file, then output as binary and Gaussian bar plots. Gaussian data are reverse log-transformed if they were zero inflated and thus log-normalised earlier, using Duan’s Smearing Estimator [51]. Binary and Gaussian data are then multiplied to give a single index of predicted abundance which is saved as a csv file twice: once alongside all of the explanatory variables and once alongside just the cell centroid latitude and longitudes. The binary and Gaussian model objects are saved—these can be loaded back into *R* for re-processing outputs and additional analysis later. A report of all model metrics, which allow the model performance to be quantified, is also saved as a csv file (see S1 File for more).

**Fig 2:**
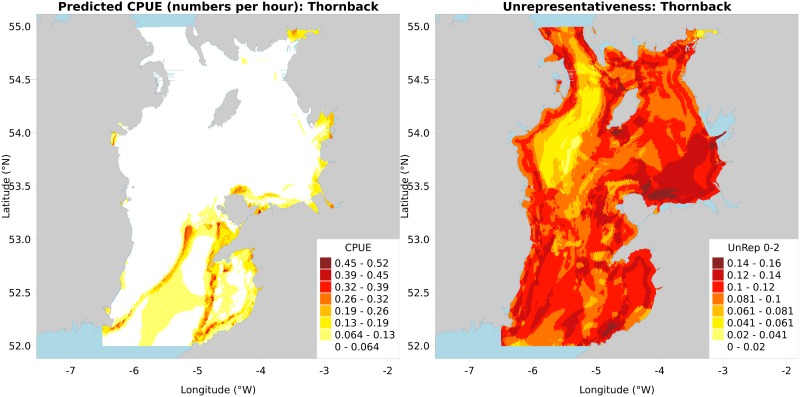
(A) Predicted CPUE map, from *gbm*.*auto*. (B) Representativeness Surface Builder map, from *gbm*.*auto*.

The predictions are then mapped, followed by the representativeness surface builder maps (Fig 2A and 2B respectively) for binary, then Gaussian, then both combined, in colour then greyscale.

Here we run the *gbm*.*auto* function with all option parameters left at their default values. Explanatory variable columns are 4 to 9 and 11; the response variable column is 12.

gbm.auto(grids = mygrids, samples = mysamples, expvar = c(4:8,10), resvar = 11)

### Acquiring global coastlines with *gbm*.*basemap*

**Fig 3:**
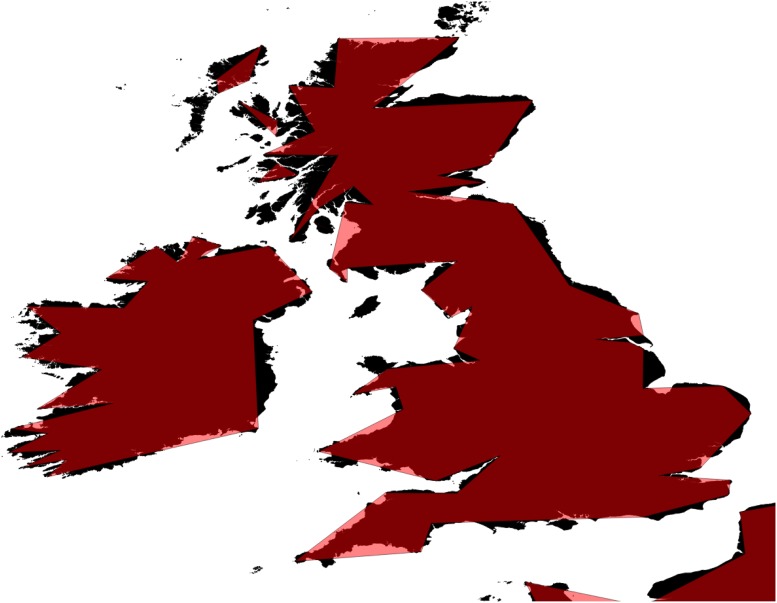
Comparison of NOAA basemaps at full (f; black, under) and coarse (c; red, over) resolution basemaps from *gbm*.*basemap*.

The acquisition and processing of coastline data at the appropriate scale and resolution is usually required for mapping, but can be time- and technically demanding. *Gbm*.*basemap* automates the acquisition and cropping of NOAA’s global coastline shapefiles database to user-defined extents, for *gbm*.*map*. This lowers memory use and processing time, and allows the user to set the map resolution. The difference in resolution between the highest (full, “f”) and the lowest (coarse, “c”) of the NOAA datasets used by *gbm*.*basemap* is shown in Fig 3 using the example of the British Isles. While the coarse dataset sacrifices a large amount of detail, this may be acceptable for large-area maps focusing more on mid-oceans than coastlines.

mymap <- gbm.basemap(grids = grids, gridslat = 2, gridslon = 1) # run the function with defaults

### Representativeness surface builder with *gbm*.*rsb*

The representativeness surface builder function compares the frequency distribution of the explanatory variables from the ‘grids’ data with those from the ‘samples’ data, summing (the modulus of) the differences into a score which indicates how well the samples data captures that variable’s full range. This is calculated for every cell in ‘grids’ and exported to a csv file, which *gbm*.*auto* has *gbm*.*map* output to a figure. Higher values signify poor coverage of those explanatory variable range sections by the samples data, and thus where users should be more cautious when drawing conclusions from the corresponding predicted abundance maps.

expvar = c(4:10) #list explanatory variable columns (as *gbm*.*auto*)

resvar = 11 #list explanatory variable columns (as *gbm*.*auto*)

rsbdf_bin <- gbm.rsb(samples = mysamples, grids = mygrids, expvarnames = names(mysamples[expvar]), gridslat = 2, gridslon = 1) # create binary data RSB

### Mapping with *gbm*.*map*

The *gbm*.*map* function handles the mapping, calculating the cell size automatically and allowing the user to alter most elements of the output. This can be run manually within R’s standard plotting framework, and is called by *gbm*.*auto*, *cons*, and *valuemap*.

data <- gbm.auto::AllPreds_E # load abundance predictions produced by *gbm*.*auto*

png(filename = paste("./Cuckoo_Map2.png",sep = ""), width = 4*1920, height = 4*1920, units = "px", pointsize = 4*48, bg = "white", res = NA, family = "", type = "cairo-png") # opens the PNG image format writing process; change type to “quartz” for non-Linux systems.

par(mar = c(3.2,3,1.3,0), las = 1, mgp = c(2.1,0.5,0),xpd = FALSE) # sets plot boundaries and sizes

gbm.map(x = data[,2], y = data[,1], z = data[,3]) # run *gbm*.*map* function

dev.off() # closes the PNG writing device, saving the image.

### Conservation mapping with *gbm*.*cons*

*Gbm*.*cons* runs *gbm*.*auto* for multiple subsets of the dataset, scaling and amalgamating the combined results, producing maps which highlight areas of high conservation importance for multiple species in the same study area. In a previous study [24] this function was used to map predicted CPUEs for juvenile and adult subsets and to locate potential nursery grounds and spawning areas. The code maps the results via *gbm*.*map* as well as saving the data as a csv file. Stepwise guides for running *gbm*.*map*, *gbm*.*basemap*, *gbm*.*rsb*, *gbm*.*loop* and *gbm*.*cons* are provided in S1 File.

mygrids <- gbm.auto::grids # load grids file

Juveniles <- gbm.auto::Juveniles # load juveniles subset

Adult_Females <- gbm.auto::Adult_Females # load adult females subset

gbm.cons(mygrids = mygrids, subsets = c("Juveniles", "Adult_Females"),

resvars = c(43:44,10:11), expvars = list(c(4:10,14,16,20,24,28,36), c(4:10,14,17,21,25,29,37), 4:9, 4:9), tcs = list(c(2,13), c(2,13), c(2,6), c(2,6)), lrs = list(c(0.01,0.005), c(0.01,0.005), 0.0001, 0.0005), zeroes = rep(FALSE,4)) # 4 entries, 4 *gbm*.*auto* runs, small lrs fail for the more data-limited subsets and were removed, zero category removed for maps, all other arguments omitted and default to their *gbm*.*auto* defaults (mostly TRUE).

### Closed area generation with *gbm*.*valuemap*

Once the predicted abundance maps have been produced by *gbm*.*map* in *gbm*.*auto*, *gbm*.*valuemap* is the DST that can generate MPA options using those species’ predicted abundance maps. Effort displacement was chosen as the value to minimise since it is the classic problem of fisheries management [16,41,52], but other proxies for the preferences of that stakeholder group (e.g. CPUE, profit) or any other stakeholder group (e.g. windfarms, fossil fuel or aggregate extraction) can be used instead—see Reference [29] for further details. The conservation value metric used by this function is based on the MSY principle of *escapement biomass*. This is the percentage of the stock which must be retained each year to conserve it, expressed as Harvest Rate at MSY (HR_MSY_). In the S1 File we generate closed areas under four different scenarios, to demonstrate the effect of different weighting choices.

**Fig 4:**
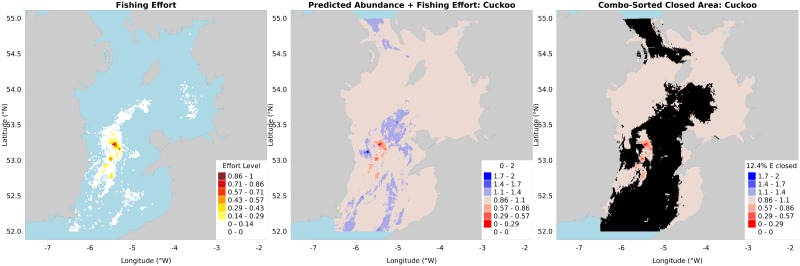
(A) Fishing effort map, from *gbm*.*valuemap*; (B) Predicted CPUE of cuckoo ray plus reversed fishing effort map, from *gbm*.*valuemap*; (C) Predicted CPUE of cuckoo ray plus reversed fishing effort map, with overlaid closed area, from *gbm*.*valuemap*.

*Gbm*.*valuemap* scales the response variable data based on importance ratios specified by the user, maps a user-defined explanatory variable to be avoided, e.g. fishing effort (Fig 4A), then combines the response variable data (e.g. cuckoo ray CPUE) with *reversed* avoid-variable data (fishing effort), both scaled to 1. This results in a combined map ranging from areas to avoid closing (value 0: maximum high fishing effort and no cuckoo ray CPUE) to areas to preferentially close (value 1: no fishing effort and maximum cuckoo ray CPUE)(Fig 4B). The data are then sorted by one of four sorting schemes and the rows summed until B*pa*—the precautionary biomass required to protect the spawning stock—is reached. These rows correspond to a closed area / MPA candidate map for each (combination sort example in Fig 4C).

**Fig 5:**
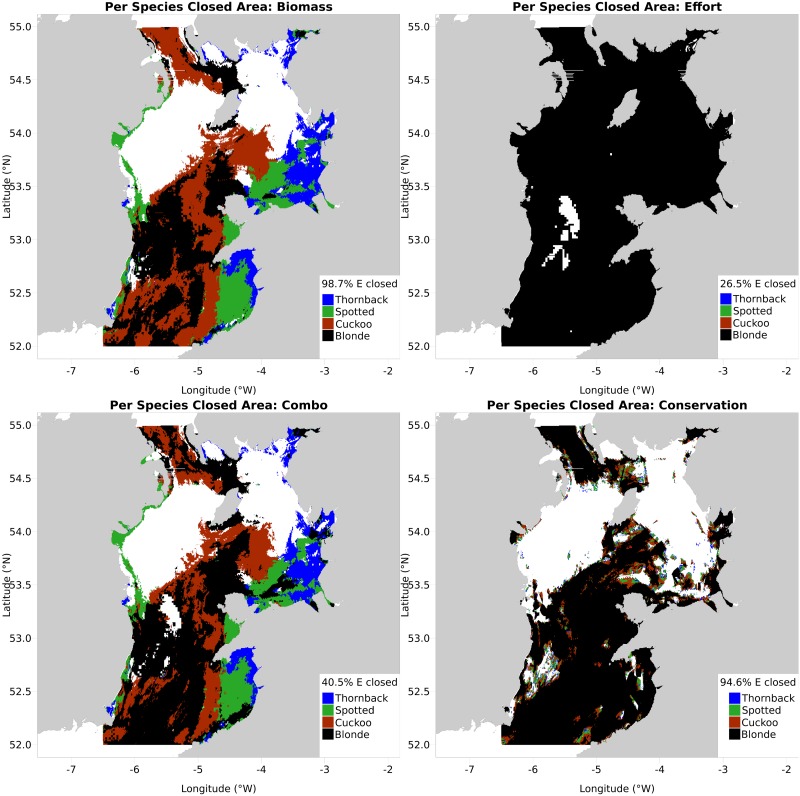
Cumulative area closure maps derived under the biomass (top left), effort (top right), combination (bottom left) and conservation (bottom right) sorting techniques, from *gbm*.*valuemap*.

The code then builds a *growing* MPA, starting with MPA map for the most conservationally at-risk species (as set by the user), then counting those MPA data rows against the *next* species, before beginning that species’ B*pa* cumulative sum. In essence, this is asking ‘how much of blonde ray’s B*pa* is already protected by the cuckoo ray MPA?’. This results in one MPA map per species, and a single four-colour MPA map, with colours corresponding to the species responsible for that part of the MPA (four-species cumulative closure maps for all four sorting scenarios shown in Fig 5). All maps generated by *gbm*.*valuemap* list the percentage of the avoid-variable’s total that is overlapped by the MPA, in the map legend. Finally a report is produced (see S1 File).

The sorting strategies mentioned earlier are as follows. The first is the combination metric as previously discussed (‘combination sort’), combining the response variable (ray CPUE) with reversed avoid-variable (fishing effort). The second (‘biomass sort’) is response variable only, preferentially protecting the areas of highest CPUE with no regard for fishing effort. The third is the opposite, preferentially avoiding fishing effort reductions (‘effort sort’). The fourth is a conservation sort, preferentially closing areas of high nursery area or spawning ground CPUE based on the conservation map produced by *gbm*.*cons*, ignoring fishing effort like the biomass sort.

conserve <- gbm.auto::AllScaledData # load data from *gbm*.*cons*.

mydata <- gbm.auto::AllPreds_E # load dataset with latitude, longitude, fishing fleet effort and all four rays’ predicted CPUE.

mydata <- cbind(mydata, conserve = conserve[,3]) #add conservation data from *gbm*.*cons* as a column to *mydata*.

To run *gbm*.*valuemap* with standard weightings and known HR_MSY_ values:

gbm.valuemap(dbase = mydata, loncolno = 2, latcolno = 1, goodcols = c(3,5,6,4), badcols = 7, conservecol = 8, HRMSY = c(0.08,0.14,0.08,0.15))

### Calculating the coefficient of variation of predicted abundance with *gbm*.*loop*

*Gbm*.*loop* repeats exactly the same *gbm*.*auto* run a user-specified number of times and collates the multiple outputs. Before the prediction stage it calculates the minimum, average, maximum, and variance of the variable influence values as seen in the bar plots, as well as plotting the minimum, average, and maximum partial dependence values for each x value in the variable’s range, for the partial dependence line plots. After the prediction stage it calculates the coefficient of variation for the predicted abundance surface, i.e. the variance of values at each cell in ‘grids’. Map and csv files are produced, enabling users to further quantify the robustness of their predictions.

gbmlooptest <- gbm.loop(loops = 5, grids = mygrids, samples = mysamples, expvar = c(4:10), resvar = 11, simp = F) # Run code with most defaults.

## Overview and benefits of *gbm*.*auto*

The *gbm*.*auto* suite of functions develop the BRT functions previously available in R’s ‘CRAN’ repository into an easily-usable and feature-rich resource primarily for fisheries, scientists to disseminate to managers and stakeholders, putting the power of these powerful mathematical tools into the hands of those who need their answers most. The package allows users to easily produce predicted abundance maps, explanatory variable diagnoses, conservation priority area maps and area closure proposals, with little work or prior knowledge required. This can facilitate and expedite fisheries managers’ jobs of conserving data-poor species using MPAs that balance competing priorities with the full engagement of stakeholders.

The ability to run subfunctions separately and reload saved model objects allows users to adjust and re-run sections of the analyses without having to re-run the whole BRT modelling process. The ability to switch off most elements in the functions (e.g. producing maps, saving data) means users can reduce analyses to the essentials they require. The default formatting, plus customisation options, allow users to quickly generate high quality outputs from these functions (*gbm*.*map*, *gbm*.*cons*, *gbm*.*valuemap*) for use in presentations and academic journals, without the need for lengthy or repeated formatting. Together these options can save users much time, accelerating the management process.

Here we have demonstrated the use of the *gbm*.*auto* package to map predicted abundances for four Irish Sea ray species, predicated on environmental and human inputs, including mapping nursery area and spawning ground candidates with *gbm*.*cons* (see S1 File), and finally resulting in MPA prediction maps under four scenarios with *gbm*.*valuemap*. These output maps and their complementary variables plots can drive collaborative MPA siting discussions with stakeholders and fisheries managers, leading to biologically-underpinned MPA proposals that have the full buy-in of the impacted industry. Such discussions should also include the range of diagnostic tools provided by *gbm*.*auto* in order to assess the strength and representativeness of the outputs, such as the RSB maps, coefficient of variation map, and model reports. This software is generalizable to other fields, other areas of marine biology most intuitively, but conceptually any spatially distributed abundance data one wishes to predictively map based on associated variables.

The main improvement scheduled for the *gbm*.*auto* package is to complete the design and build of a JavaScript frontend that will incorporate the outputs of the *gbm*.*auto* package and allow stakeholders to design their own closed areas, with displayed levels of species conservation dynamically changing as their designs evolve. This will allow stakeholders to propose MPAs underpinned by their own preferences and harvest-rate fisheries science. They could do this alone or collaboratively as a collective of fishermen, handled within the existing management framework and leading to a scientist/manager/stakeholder discussion as normal. This would increase stakeholder buy-in and allow the industry a greater degree of autonomy. In addition, many processing speed increases, completion time estimates, argument auto-optimisation, and similar performance improvements are planned.

The hope is that this software becomes established as the standard tool for scientists to conduct spatial predictions using BRTs in R. We intend to submit the package to the CRAN repository, and continue to develop and maintain it there and on GitHub, where collaborations are fuelling its on-going development.

## Conclusion

The novel tool we have built and showcase here greatly simplifies the process of predictively mapping the distribution of species and their subsets using powerful machine-learning mathematics, without the need for rich datasets. Socioeconomic costs and harvest-rate data are then integrated into a closed-area generating decision support tool that allows (fisheries) scientists and managers to fully involve stakeholders in the conservation processes. This tool facilitates the use of powerful BRT mapping to assess and manage single species as well as multiple species and subsets collectively, assisting practitioners and benefiting managers with a rich suite of graphical outputs and statistical results. By making spatial BRT analysis more readily available to the ecological- and wider scientific community, we anticipate that adoption of this approach will grow, improving the quality of spatial prediction analyses.

## Supporting information

S1 File A supplementary document explaining the running of the code in more detail, listing the functions’ arguments and their usage, a guide to running the functions together and separately, as well as additional results outputs, accompanies this manuscript. (PDF)

S2 File A short document detailing the data sources and their processing techniques. (PDF)

S3 File R functions and packages used. (PDF)

## Abbreviations

B*pa*: Precautionary reference point for spawning stock biomass
BRT: Boosted Regression Tree
CPUE: Catch Per Unit Effort
DST: Decision Support Tool
GAM: Generalised Additive Modelling
GLM: Generalised Linear Modelling
HR: Harvest Rate
MARXAN: Marine spatially Explicit Annealing
MaxEnt: Maximum Entropy
MPA: Marine Protected Area
MSY: Maximum Sustainable Yield
