# Correction: Gbm.auto: A software tool to simplify spatial modelling and Marine Protected Area planning

**DOI:** 10.1371/journal.pone.0192520

**Published:** 2018-02-02

**Authors:** Simon Dedman, Rick Officer, Maurice Clarke, David G. Reid, Deirdre Brophy

The subheading “Worked examplePre-run parameter scoping with gbm.bfcheck” under the Guide to software functions section is incorrect. The subheadings should be “Worked example” and “Pre-run parameter scoping with gbm.bfcheck”.

The following information is missing from the eighth paragraph of the Guide to software functions section under the subheading “Worked example”:

To demonstrate *gbm*.*auto* we will run a simple example in the sections below; here we load the required functions and data. Please see Supplementary Material D1 for all details of each function and more fully worked examples.

install.packages("devtools")

library("devtools")

install_github('SimonDedman/gbm.auto')

library("gbm.auto")

mygrids <- gbm.auto::grids # load grids

mysamples <- gbm.auto::samples # load samples
