# Supplementary material for: Gbm.auto: A software tool to simplify spatial modelling and Marine Protected Area planning
Source: PLoS One. 2017 Dec 7;12(12):e0188955. doi: 10.1371/journal.pone.0188955 (PMC5720763; doi:10.1371/journal.pone.0188955)
Supplement: S1 File — (PDF) [file pone.0188955.s001.pdf]

Gbm.auto: a software tool to simplify spatial modelling and Marine Protected Area planning

Simon Dedman[1,2*], Rick Officer[1], Maurice Clarke[2], David G. Reid[2], Deirdre Brophy[1]

[1] Marine and Freshwater Research Centre, Galway-Mayo Institute of Technology, Galway, Ireland; +1 415 944 7258

[2] Marine Institute, Rinville, Oranmore, Co. Galway, Ireland

* Corresponding author

Email: simondedman@gmail.com (SD)

Supplementary Material Appendix S1

Below follows the model arguments for *gbm.auto* and its subfunctions, and a guide for running *gbm.auto* then the subfunctions independently.

1. What *gbm.auto* does and how – additional detail

With the working directory and variable columns set by the user, the code loads all required functions then begins looping through the arguments provided by the user. This begins with the response variable, which can be greater than one, e.g. the four ray species modelled in [1,2]. If the user has asked the model to check whether their data are zero inflated, the code performs a simple test to see if over half of the data are zeroes, marking the data zero inflated if so. The code then checks to ensure the data has any zeroes at all – this method assumes the samples data will be a mixture of successful and unsuccessful trials – and warns the users if otherwise. A vector of binary data are then created from the (Gaussian) samples data, by converting all non-zero values to 1. If data are zero inflated, they are then log-transformed to better conform to the expectation of a Gaussian distribution.

The remaining argument loops are then begun, with bag fraction nested within learning rate nested within tree complexity. Assuming the data are zero inflated, the first binomial BRT is then launched, and its testing/training correlation scores logged in a container, along with the combination of arguments associated with that BRT run; the first Gaussian BRT is run next, under the same conditions. Both BRTs' detailed results metrics are saved into a report table, then the end of the argument loops is reached and the code moves to the next loop. For each loop, the performance scores for the binary and Gaussian BRTs are compared to the scores in the 'best' container, ousting it if they improve upon it.

The best model combination is chosen based on internal performance metrics that are generated by the model, such as the training data correlation (higher is better) and mean deviance (lower is better). Performance of the best binary models was quantified using the Area Under the Receiver-Operator-Characteristic Curve (AUC) statistic [3]. This is calculated for the training data and the cross-validated (cv) data used in the model; the size of discrepancy between the two indicates overfitting. The BRT modelling process is designed to reduce overfitting via the cross-validation subroutine [4].

Next, *gbm.simplify* is run on both 'best' models to see if better results can be attained for either by dropping unnecessary variables. If they can, the simplified version of that BRT is retained in the 'best' model container.

With the best binary and Gaussian models achieved, the code produces the results. Line plots of partial deviance are created, first all on one plot, then for each variable separately, for binary and Gaussian as usual. Next are dot plots of the spread of partial deviance against the explanatory variable values. Finally the influence of each variable's contribution to the model is tabulated and saved as a csv, then output as a bar plot.

After these intermediary visual outputs are produced, the size of the interactions between the explanatory variables is calculated, before moving onto the final stage: the predictions and mapping. As normal, binomial data are handled first, again only if the data are zero inflated, with predictions handled by *gbm.predict.grids*. Gaussian data are handled next, after first being back-transformed – if they were zero inflated and thus log-normalised earlier – using Duan's Smearing Estimator [5]. Binary (the likelihood of a positive sample; 0 or 1) and Gaussian (the probable number of samples) data are then multiplied to give a single index of predicted abundance. These predictions are saved as a gridded csv file, twice: once alongside all of the explanatory variables for each grid cell, and once with just the cell centroid latitudes and longitudes. The report table of all model metrics is updated and saved as a csv file.

The predictions are then mapped. First in colour and then greyscale, and then the representativeness surface builder maps are generated for both binary, then Gaussian data, then both combined – again in colour then greyscale.

2. Function Arguments

The function arguments are as follows:

gbm.bfcheck

- 'samples', 'resvar' and 'ZI': see explanation in *gbm.auto* section below.

gbm.auto

- 'samples' as mentioned previously, contains the response variable and predictor variables;. Column names should be short and simple, shouldn't start with numbers nor end with periods, and cannot contain spaces. The column name of the response variable will be used in map titles elsewhere, so should ideally be the species name, e.g. WhiteShark.

- 'grids' is the explanatory data that will be used to predict species presence /absence or abundance at new sites where the response variable was not recorded. The names of the columns for the explanatory data must match those in 'samples'. Data are imported with read.csv (for csv files); it defaults to NULL, which is blank as detailed above. Data are expected to be gridded i.e. points in a repeating pattern equidistant from their horizontal and vertical neighbours. 'grids' data not in this format may cause *gbm.map* to fail, or to produce unexpected results. Abundance predictions are still generated correctly, however, and can be mapped separately.
- 'expvar' is a list of the column numbers in 'samples' that contain the explanatory variables, using R's concatenate function. They need not be contiguous, e.g. c(1,3,5,8) is acceptable.
- 'resvar' is the column number(s) of the response variable(s) in 'samples'. Setting a vector of values will cause the model to loop through the entire process for each response variable, outputting the results into response-variable-specific subfolders (e.g. species names) within the working directory. For delta log-normal BRTs, these data should contain zeroes.
- 'tc' is the list of permutations of **tree complexity** allowed, related to the number of explanatory variables; defaults to a vector of 2 and the number of explanatory variables, i.e. only two-variable interactions are modelled, then all variables are modelled interacting simultaneously. Higher tc numbers take an increasingly long to time to model.
- 'lr' is the list of permutations of **learning rate** to be tested, with smaller rates taking longer to process but being more likely to produce a viable result with limited data. Default is c(0.01,0.005).
- 'bf' is the list of permutations of **bag fraction** allowed. Default is 0.5. See 'Arguments for *gbm.bfcheck*' section for details on optimising this value.
- 'ZI' tests for **zero-inflated** data, common in fisheries. Accepted inputs are TRUE if you know the data are zero-inflated, FALSE if you know they are not, and "CHECK" to perform a crude test to determine this before proceeding. The outcome will be in the

final report. Default is "CHECK"; note the R syntax whereby TRUE and FALSE have no quotation marks whereas "CHECK" does.

- 'fam1' controls the probability distribution family for first part of delta process, defaulting to "bernoulli".
- 'fam2' controls the probability distribution family for second part of delta process, defaulting to "gaussian".
- 'simp' allows the user to disable *gbm.simplify*, a function which tests whether the BRTs perform better with variables omitted. If BRTs are struggling to run, for example due to sparse data, trying to simplify them can further reduce available information and cause the BRTs to fail to run. Default is TRUE.
- 'gridslat' is the column number of latitude in 'grids'; default is 2.
- 'gridslon' is the column number of longitude in 'grids'; default is 1.
- 'cols' is an optional vector of colours filling the bar plots, one per explanatory variable, causing bars for specific explanatory variables to be of uniform colour in all output graphics. Default is grey.colors(1,1,1) which is white bars; since the variable name is printed in the bar the colours may be unnecessary for small numbers of variables.
- 'linesfiles' exports line plot data to individual csv files, which can be used as functional response curve data for EcoSpace models in EcoPath with EcoSim. Default 'FALSE'.
- 'smooth' applies a smoother to the partial dependence line plots; default is 'FALSE'.
- 'savegbm' controls whether the model object is saved to the active directory. This allows it to be reloaded later with load("objectname"), avoiding the need to re-run what can be very time-consuming processing jobs, before re-doing post-processing jobs. The downside is that the object can be large and running multiple repeat iterations can consume increasing disk space. Options are TRUE or FALSE, default is TRUE.
- 'varint' allows users to switch off **variable interaction** calculations and their subsequent reporting. In extremely data-poor situations this element can fail, causing the whole process to fail, so making varint FALSE allows one to proceed. Default is TRUE.

- ‘map’ controls whether predicted abundance maps are generated; default TRUE.
- ‘shape’ sets the coast shapefile. Format is expected to be a shapefile list object, as created by *read.shapefile* from the ‘shapefiles’ package, e.g. MyMap <- read.shapefile(“Map”). Default is NULL which results in the shapefile being automatically generated for your data using *gbm.basemap*.
- ‘RSB’ controls whether maps of the representativeness of the predicted abundance map are generated using the **Representativeness Surface Builder** tool; default TRUE.
- ‘BnW’ controls whether to repeat maps (including RSB maps) in **black and white** for print journals; default TRUE.
- ‘alerts’ plays notification sounds to alert the user when the code reaches key stages, fails, or completes; defaults to TRUE.
- ‘pngtype’ allows the user to change the graphical device used to generate the png images. Defaults to “cairo-png”, try “quartz” (especially for Apple OSX) or “Xlib” if no graphics are produced.
- ‘gaus’ controls whether to process the second part of delta process. Default TRUE, if FALSE only the first part will be run. Useful for very highly zero-inflated data where the presence-only subset is so small that BRT runs may not run.
- … optional to be passed to subordinate functions, notably *zero* in *breaks.grid* in *gbm.map*, *legend* in *legend.grid* in *gbm.map*, and *gbm.step* arguments such as *n.trees*.

gbm.basemap

- ‘bounds’, the region to crop to, format is c(xmin,xmax,ymin,ymax).
- ‘getzip’, whether to download and unpack NOAA’s world coastline shapefile data; default is TRUE, otherwise enter the relative or absolute location to the GSHHS_shp folder created by unzipping the NOAA zip file, including naming the folder.
- ‘zipvers’, enables the database version to be changed in the event that NOAA update is. Defaults to “2.3.5-1” (as of *gbm.auto* version 1.0.4).

- ‘savename’, the filenames for the exported shapefiles, with no extension. Defaults to “Crop_Map”.
- ‘res’, resolution, 1:5 (low to high) or “c”, “l”, “i”, “h”, “f” for coarse, low, intermediate, high, full, or “CALC” to automatically calculate an appropriate resolution based on the maximum extents of the bounds supplied.
- ‘extrabounds’, Grow bounds 16pct each direction to expand rectangular datasets’ basemaps over the entire square area created by basemap in mapplots. Defaults to FALSE.

gbm.rsb

- ‘samples’, a data frame with response and explanatory variables.
- ‘grids’, a data frame of (more/different) explanatory variables & no response variable, to be predicted to by *gbm.predict.grids*.
- ‘expvarnames’, a vector of column names of explanatory variables being tested. Can be of length 1. Names must match in ‘samples’ and ‘grids’.
- ‘gridslat’, the column number for latitude in ‘grids’.
- ‘gridslon’, the column number for longitude in ‘grids’.

gbm.map

- ‘x’ is the vector of longitudes, from *make.grid* in *mapplots*. Ideally order your data by this, descending, second.
- ‘y’ is the vector of latitudes, from *make.grid* in *mapplots*. Order by this, descending, first,
- ‘z’ is the vector of abundances generated by *gbm.predict.grids*, from *make.grid* in *mapplots* (grids[,predabund]).
- ‘byx’ and ‘byy’, set longitudinal width and latitudinal height of the grid cells in *make.grid*; defaults to being auto-calculated by *gbm.map*.
- ‘grdfun’, is the mathematical operation performed in unexpected cases where there are >1 data points per cell. Defaults to *mean*; other vector operations are acceptable,

e.g. *sum, prod, min, max, sd, se, var* (use the last 3 with caution: the *gbm.auto* / *gbm.map* functions calling these will typically have 1 or very few values per grid cell).

- ‘mapmain’, sets the start of the predicted abundance map’s title, to which the response variable name is appended. Defaults to “Predicted Abundance: ”.
- ‘species’, from *basemap* in *mapplots*; the response variable name. Defaults to "Response Variable" when *gbm.map* is used alone; *gbm.auto* sets it to the name of the current species in the loop when calling *gbm.map* as part of its routine.
- ‘heatcolours’, is the abundance colour scale. Defaults to yellow-to-red heat colours from *mapplots*.
- ‘colournumber’, is the number of colours to spread ‘heatcolours’ over, defaulting to eight, which reveals small enough differences within the results without creating an over-large legend.
- ‘shape’, is the base map shape to draw. Format is expected to be a shapefile list object, as created by *read.shapefile* from the ‘shapefiles’ package, e.g. MyMap <- read.shapefile(“Map”). Default is NULL which results in the shapefile being automatically generated for your data using *gbm.basemap*.
- ‘landcol’, is the colour for the null area of the map, which is land for marine plots. Defaults to “darkgreen”.
- ‘mapback’, controls the background colour of the map. Defaults to “lightblue”, for marine plots.
- ‘legendloc’, for *legend.grid*, controls the location of the legend on the map; defaults to “bottomright”, can be either a named position or a 2D vector in the form c(x,y); see the *legend* package manual for more details.
- ‘legendtitle’, for *legend.grid*, is the metric of abundance used in the legend title in the predicted abundance maps and representativeness maps; defaults to “CPUE”.
- ‘lejback’, for *legend.grid*, is the background colour of the legend. Defaults to “white”.
- ‘zero’, for *breaks.grid*, controls whether to include a zero-only category in the colour breakpoints and subsequent legend. Defaults to TRUE.

- ‘quantile’, for *breaks.grid*, sets the maximum value of the breakpoints. Defaults to 1 (i.e. 100%: all values); lower this to cut off high outliers.
- ‘byxout’ exports ‘byx’ for use elsewhere. g*bm.valuemap* uses this to maintain grid cell size for closed area overlays. Default is FALSE.
- ‘breaks’ is a vector of breakpoints for colour scales. Defaults to NULL, leading them to be created automatically.
- ‘…’ Additional arguments for *legend.grid*'s ‘...’, which passes to *legend.*

gbm.cons

- ‘mygrids’, the csv file (including its location relative to the current folder) of gridded data to predict to. No default.
- ‘subsets’, a single item or vector of subset name(s), corresponding to samples dataset objects already read into the environment, e.g. using read.csv().
- ‘alerts’, ‘map’ and ‘BnW’, as per *gbm.auto*.
- ‘expvars’, a list object of *expvar* vectors for *gbm.auto* runs, with number of list items equal to the number of subsets multiplied by the number of species, i.e. the total number of *gbm.auto*s to be run. No default.
- ‘resvars’, a vector of *resvar* column numbers in the *conssamples* files for *gbm.auto* runs, length per the number of list items for *expvars*, above. No default.
- ‘gbmautos’: do *gbm.auto* runs for species? Default TRUE; set to FALSE if they have already run and their output files are in the expected directory locations.
- ‘tcs’, ‘lrs’, ‘bfs’, ‘ZIs’, ‘colss’, ‘linesfiless’, ‘savegbms’, ‘varints’, ‘maps’, ‘RSBs’, ‘BnWs’, ‘zeroes’: pluralised versions of *gbm.auto* arguments, with lists (‘tsc’, ‘lrs’, ‘colss’) or vectors (the rest) the same length as ‘expvars’ and ‘resvars’. Note all are the *gbm.auto* arguments with a terminal ‘s’ except ‘zero’ which is ‘zeroes’. All default to the *gbm.auto* defaults.
- ‘shape’, the coastline shapefile underpinning the generated maps, defaults to generating a basemap from the extents of ‘dbase’, using *gbm.basemap*. User-specific alternatives can be created manually with *gbm.basemap*.

- ‘pngtype’ allows the user to change the graphical device used to generate the png images. Defaults to “cairo-png”, try “quartz” (especially for Apple OSX) or “Xlib” if no graphics are produced.
- ‘gridslat’ and ‘gridslon’ per *gbm.auto*, defaulting to 2 and 1 respectively.

gbm.valuemap

- ‘dbase’, the data frame to load; it expects longitude, latitude and data columns: predicted abundances, fishing effort, and so forth. The *gbm.auto* csv outputs satisfy this condition; append the predicted abundance columns from multiple species onto one sheet (see the ‘AllPreds_E’ dataset for an example).
- ‘loncolno’, the column number in ‘dbase’ which has longitudes; default is 1.
- ‘latcolno’, the column number in ‘dbase’ which has latitudes; default is 2.
- ‘goodcols’, the column numbers with *gbm.*auto predicted abundances, where higher is better, listed in descending order of conservation importance e.g. c(3,1,2,4).
- ‘badcols’, the column numbers with stressor elements e.g. fishing, where higher is worse.
- ‘conservecol’, the conservation column, from *gbm.cons*. Defaults to NULL.
- ‘HRMSY’, the maximum percentage of each ‘goodcols’ stock which can be removed each year, as a decimal e.g. 0.15 = 15%. A single number or vector with the same order as ‘goodcols’. Default is NULL.
- ‘plotthis’, which sections to run and graphics to plot, defaults to all. In the order they are processed, “bad” produces the stressor map e.g. fishing effort – this can be produced separately with *gbm.map* directly. “good” produces the abundance elements e.g. CPUE, that should have already been plotted when the *gbm.auto*s were run to create the predicted abundance data used by ‘dbase’. “both” maps the combination of bad and good, but also generates a cell size value used by “close” later. If running “close” it’s advisable to also run “both”. “close” generates all the closed area data and maps, the primary point of the function. The user can turn individual sections off by

removing them from this argument; to delete them all set it to NULL. Default: c("good","bad","both","close").

- ‘maploops’, which sorting loops to run. Can remove any of them, defaults to c("Combo","Biomass","Effort","Conservation").
- ‘savethis’, exports all data as a CSV file; defaults TRUE.
- ‘HRMSY’ is the maximum percent of each ‘goodcols’ stock which can be removed yearly, as a decimal (0.15 = 15%). The remainder which must be protected is 1-HRMSY. A single number or vector, in the same order as ‘goodcols’.
- ‘goodweight’, one or a vector of weighting multiple(s) for ‘goodcols’ array; default is NULL.
- ‘badweight’, one or a vector of weighting multiple(s) for ‘badcols’ array, no default is NULL.
- ‘m’, the multiplication factor for Bpa units, default is 1. Use 1000 to convert tonnes to kilos, 0.001 kilos to tonnes. Assumed to be the same for all ‘goodcols’.
- ‘alerts’, as per *gbm.auto*, defaulting to TRUE.
- ‘BnW’, as per *gbm.auto*, defaulting to TRUE.
- ‘shape’, the coastline shapefile underpinning the generated maps, defaults to generating a basemap from the extents of ‘dbase’, using *gbm.basemap*. User-specific alternatives can be created manually with *gbm.basemap*.
- ‘pngtype’ allows the user to change the graphical device used to generate the png images. Defaults to “cairo-png”, try “quartz” (especially for Apple OSX) or “Xlib” if no graphics are produced.
- ... optional arguments for *gbm.map*.

gbm.loop

While various performance metrics from the BRT modelling process are included in the report csv and the RSB map, there is no way to estimate a measure of the coefficient of variation of the predicted abundances natively within the BRT modelling process. *Gbm.loop* addresses this issue by repeating the same *gbm.auto* arguments a user-set

number of times, and calculating the coefficient of variation for each sample site in the study area, resulting in csv files of the output, and a map (Fig 6). Its arguments are:

- 'loops' is an integer defining the number of repetitions of the *gbm.auto* arguments to run.
- 'savecsv' is a TRUE/FALSE option defining whether or not to produce csv files of the outputs.
- 'calcpreds' should coefficients of variation of predicted abundance be calculated?
- 'varmap' is a TRUE/FALSE option defining whether or not to produce a map of the outputs.
- 'measure' is a text field, essentially 'the coefficient of variation of what?'. Defaults to CPUE.
- 'cleanup' is a TRUE/FALSE option defining whether or not to delete the *gbm.auto*-created folders once their abundance predictions have been used.
- All other arguments (including shape for pre-setting the map background) are as per *gbm.auto*, though note 'linesfiles' defaults to TRUE; the code will likely fail otherwise.

3. Stepwise function runs

Functions *gbm.map*, *gbm.basemap*, *gbm.rsb*, *gbm.cons, gbm.bfcheck, gbm.loop,* and *gbm.valuemap* can be run independently after the full model has been run and generated the "Abundance_Preds_Only.csv" file, or on raw or new data.

To load the sample data and required functions, open R or RStudio and input the following code; "#" precedes comments not read by the program. Re-run the final two commands for each new session.

```
install.packages("devtools")
library("devtools")
install_github('SimonDedman/gbm.auto')
library(gbm.auto)  # load the gbm.auto suite
setwd("C:/Users/…/BRT")  # reset the working directory if required.
```

gbm.bfcheck

`mysamples <- gbm.auto::samples` # load samples

`gbm.bfcheck(samples = mysamples, resvar = 11)` # Run code with defaults

gbm.auto

Here we run the *gbm.auto* function with all option arguments left at their default values. Explanatory variable columns are 4 to 9 and 11; the response variable column is 12.

`mygrids <- gbm.auto::grids` # load grids

`mysamples <- gbm.auto::samples` # load samples

```
gbm.auto(grids = mygrids, samples = mysamples, expvar = c(4:8,10), resvar =
11)
```

gbm.basemap

`grids <- gbm.auto::grids` # read in grids file

users can then manually specify the bounding area desired:

`gridslat = 2` # label the latitude and longitude columns

`gridslon = 1`

`bounds <- c(range(grids[,gridslon]), range(grids[,gridslat]))` # set the x and y bounds

run the function with defaults for ‘getzip’ (TRUE: will download the zip file), ‘zipvers’, ‘savename’ and ‘res’ (“CALC”: will calculate the best resolution):

`mymap <- gbm.basemap(bounds = bounds)`

Or feed the grids database and its latitude and longitude columns directly to *gbm.basemap*:

`mymap <- gbm.basemap(grids = grids, gridslat = 2, gridslon = 1)`

gbm.rsb

In this example we'll generate RSB maps for binary, Gaussian, and both together, for cuckoo ray only. *Gbm.auto* does this automatically if instructed. Begin by setting the working directory and loading the *gbm.auto* package, grids, and samples as before.

`expvar = c(4:10)` #list explanatory variable columns (as *gbm.auto*)

`resvar = 11` #list explanatory variable columns (as *gbm.auto*)

`rsbdf_bin <- gbm.rsb(samples = mysamples, grids = mygrids, expvarnames = names(mysamples[expvar]), gridslat = 2, gridslon = 1)` # create the RSB data for binary data

`mysamples$brv <- ifelse(mysamples[11] > 0, 1, 0)` # create a subset of only positive data for Gaussian

`pos_samples <- subset(mysamples, brv >0)`

`rsbdf_gaus <- gbm.rsb(samples = pos_samples, grids = grids, names(mysamples[expvar]), gridslat = 2, gridslon = 1)` # create the RSB data for binary data

Map the binary RSB data in colour for cuckoo ray

```
png(filename = "Cuckoo_RSB_Bin.png", width = 4*1920, height = 4*1920, units
= "px", pointsize = 4*48, bg = "white", res = NA, family = "", type =
"cairo-png")
par(mar=c(3.2,3,1.3,0), las=1, mgp=c(2.1,0.5,0),xpd=FALSE)
gbm.map(x = mygrids[,1], y = mygrids[,2], z =
rsbdf_bin[,"Unrepresentativeness"], mapmain = "Unrepresentativeness: ",
species = "Cuckoo Ray", legendtitle = "UnRep 0-1", shape = Crop_Map)
dev.off()
```

Map the Gaussian RSB data in colour for cuckoo ray

```
png(filename = "Cuckoo_RSB_Gaus.png", width = 4*1920, height = 4*1920,
units = "px", pointsize = 4*48, bg = "white", res = NA, family = "", type =
"cairo-png")
par(mar=c(3.2,3,1.3,0), las=1, mgp=c(2.1,0.5,0),xpd=FALSE)
gbm.map(x = mygrids[,1], y = mygrids[,2], z =
rsbdf_gaus[,"Unrepresentativeness"], mapmain = "Unrepresentativeness: ",
species = "Cuckoo Ray", legendtitle = "UnRep 0-1", shape = Crop_Map)
dev.off()
```

Map the binary and Gaussian RSB data combined in colour for cuckoo ray

```
png(filename = "Cuckoo_RSB_Both.png", width = 4*1920, height = 4*1920,
units = "px", pointsize = 4*48, bg = "white", res = NA, family = "", type =
"cairo-png")
par(mar=c(3.2,3,1.3,0), las=1, mgp=c(2.1,0.5,0),xpd=FALSE)
gbm.map(x = mygrids[,1], y = mygrids[,2], z =
rsbdf_bin[,"Unrepresentativeness"] + rsbdf_gaus[,"Unrepresentativeness"],
mapmain = "Unrepresentativeness: ", species = "Cuckoo Ray", legendtitle =
"UnRep 0-2", shape = Crop_Map)
dev.off()
```

gbm.map

`Crop_Map <- read.shapefile("./CroppedMap/Crop_Map")` # read in the cropped map background to save it being re-downloaded and processed

`setwd("./Cuckoo")` # set the working directory to the cuckoo ray output

`data <- gbm.auto::AllPreds_E` # load abundance predictions produced by *gbm.auto*

`png(filename = paste("./Cuckoo_Map2.png",sep=""), width = 4*1920, height = 4*1920, units = "px", pointsize = 4*48, bg = "white", res = NA, family = "", type = "cairo-png")` # opens the PNG image format writing process

`par(mar=c(3.2,3,1.3,0), las=1, mgp=c(2.1,0.5,0),xpd=FALSE)` # sets plot boundaries and sizes

`gbm.map(x = data[,2], y = data[,1], z = data[,3], mapmain = "CPUE: ",`

`species = "Cuckoo Ray", legendtitle = "CPUE", shape = Crop_Map)` # run *gbm.map* function

`dev.off()` # closes the PNG writing device, saving the image.

gbm.cons

In this first example we'll let *gbm.cons* run through the *gbm.auto* processing of the first two species in both of the juvenile and adult female subsets (cuckoo and thornback ray), again using the previously generated coastline shapefile. The processing is quite time-consuming hence only two species are demonstrated.

`mygrids <- gbm.auto::grids` # load grids file

`Juveniles <- gbm.auto::Juveniles` # load juveniles subset

`Adult_Females <- gbm.auto::Adult_Females` # load adult females subset

`dir.create("Cons")` # Create a subfolder called "Cons"

`setwd("./Cons")` # Set that as the working directory.

```
gbm.cons(mygrids = mygrids,
        subsets = c("Juveniles", "Adult_Females"),  #specify subset objects
        alerts = TRUE, map = TRUE, BnW = TRUE,
        resvars = c(43:44,10:11),
        gbmautos = TRUE,
        expvars = list(c(4:10,14,16,20,24,28,36),
                       c(4:10,14,17,21,25,29,37),
                       4:9, 4:9), # 4 entries, 4 gbm.auto runs
        tcs = list(c(2,13), c(2,13), c(2,6), c(2,6)),
        lrs = list(c(0.01,0.005), c(0.01,0.005), 0.0001, 0.0005), # small
    lrs fail for the more data-limited subsets and were removed
```

`zeroes = rep(FALSE,4),` # remove the zero category for maps. All other arguments are omitted and default to their *gbm.auto* defaults (mostly TRUE).

`shape = Crop_Map)` # assumed to still be in your environment

In this second run we are going to set the working directory to the directory where previous *gbm.auto* runs for these subsets have already generated the required 'Abundance_Preds_only.csv' files ("Cons"). This is useful if the full run has been interrupted, such as when a *gbm.auto* run fails because the learning rate has been set too low to resolve a small dataset. The user can manually re-run that and other remaining *gbm.auto* runs, then run the following code, omitting *gbm.auto* arguments and leaving 'map', 'BnW' and 'alerts' to default to TRUE:

```
gbm.cons(mygrids = mygrids, subsets = c("Juveniles", "Adult_Females"),
         gbmautos = FALSE, resvars = c(43:44,10:11), shape = Crop_Map)
```

gbm.valuemap

Load the *gbm.auto* package then:

`Crop_Map <- read.shapefile("./CroppedMap/Crop_Map")` # read in the cropped map background to save it being re-downloaded and processed

`dir.create("Valuemaps")` # create the 'Valuemaps' subdirectory

`setwd("Valuemaps")` # set that as the working directory.

`conserve <- gbm.auto::AllScaledData` # load data from *gbm.cons*.

`mydata <- gbm.auto::AllPreds_E` # load dataset with latitude, longitude, fishing fleet effort and all four rays' predicted CPUE.

`mydata <- cbind(mydata, conserve = conserve[,3])` #add conservation data from *gbm.cons* as a column to *mydata*.

Create four blank folders in Valuemaps: Standard (all weightings at default 1), Conserveweight 10s (all rays' weightings as 10), Fishingweight 10 (fishing effort

weighting 10), Conserveweight 4 3.5 1.5 1 (cuckoo blonde spotted and thornback rays given individual weightings based on their estimated relative conservation priority):

```
dir.create("Standard")
dir.create("Conserveweight 10s")
dir.create("Fishingweight 10")
dir.create("Conserveweight 4 3.5 1.5 1")
```

To run *gbm.valuemap* with standard weightings and known HR_{MSY} values:

`setwd("Standard")` # set working directory

```
gbm.valuemap(dbase = mydata, loncolno = 2, latcolno = 1, goodcols =
c(3,5,6,4), badcols = 7, conservecol = 8, HRMSY = c(0.08,0.14,0.08,0.15),
shape = Crop_Map)
```

To run with effort weight as 10 "Fishingweight":

Between runs you may wish to clear the workspace to free up working memory. You can do so by typing `rm(list = ls())` or by clicking the broom icon in RStudio's Environment tab; you will then need to reload the *gbm.auto* package (`require(gbm.auto)`) and run the 'conserve; mydata; mydata' lines above to reload the input data. The relative location of Crop_Map has changed so this is reflected below.

```
setwd("../Fishingweight 10")
Crop_Map <- read.shapefile("../../CroppedMap/Crop_Map") # repeat this in
later runs
gbm.valuemap(dbase = mydata, loncolno = 2, latcolno = 1, goodcols =
c(3,5,6,4), badcols = 7, conservecol = 8, HRMSY = c(0.08,0.14,0.08,0.15),
Fishingweight = 10, shape = Crop_Map)
```

To run with species weights all as 10 "Conserveweight":

```
setwd("../Conserveweight 10s")
```

```
gbm.valuemap(dbase = mydata, loncolno = 2, latcolno = 1, goodcols =
c(3,5,6,4), badcols = 7, conservecol = 8, HRMSY = c(0.08,0.14,0.08,0.15),
Conserveweight = c(10,10,10,10), shape = Crop_Map)
```

To run with species weights set individually:

```
setwd("../Conserveweight 4 3.5 1.5 1")
gbm.valuemap(dbase = mydata, loncolno = 2, latcolno = 1, goodcols =
c(3,5,6,4), badcols = 7, conservecol = 8, HRMSY = c(0.08,0.14,0.08,0.15),
Conserveweight = c(4,3.5,1.5,1), shape = Crop_Map)
```

gbm.loop

Begin by setting the working directory and loading the *gbm.auto* package as before.

```
gbmlooptest <- gbm.loop(loops = 5, cleanup = T, grids = mygrids, samples =
mysamples, expvar = c(4:10), resvar = 11, simp = F) # Run code with most
defaults
```

4. Results

The principal results are displayed and explain in the main document. In addition, below are examples of the bar plots of the size of influence of explanatory variables on the response variable (Fig 1), dot plot matrices of spread of explanatory variables (Fig 2), line plot matrices of partial influence relationships of explanatory variables on the response variable (Fig 3), and partial influence relationships of explanatory variables on the response variable (Fig 4). The multi-colour cumulative closed area maps generated by *gbm.valuemap* are presented in the main document (Fig 5); in addition, the code generates one MPA map for each species as the cumulative closure grows (Fig 5). An output map from *gbm.loop* is presented for the coefficient of variation for cuckoo ray CPUE (Fig 6).

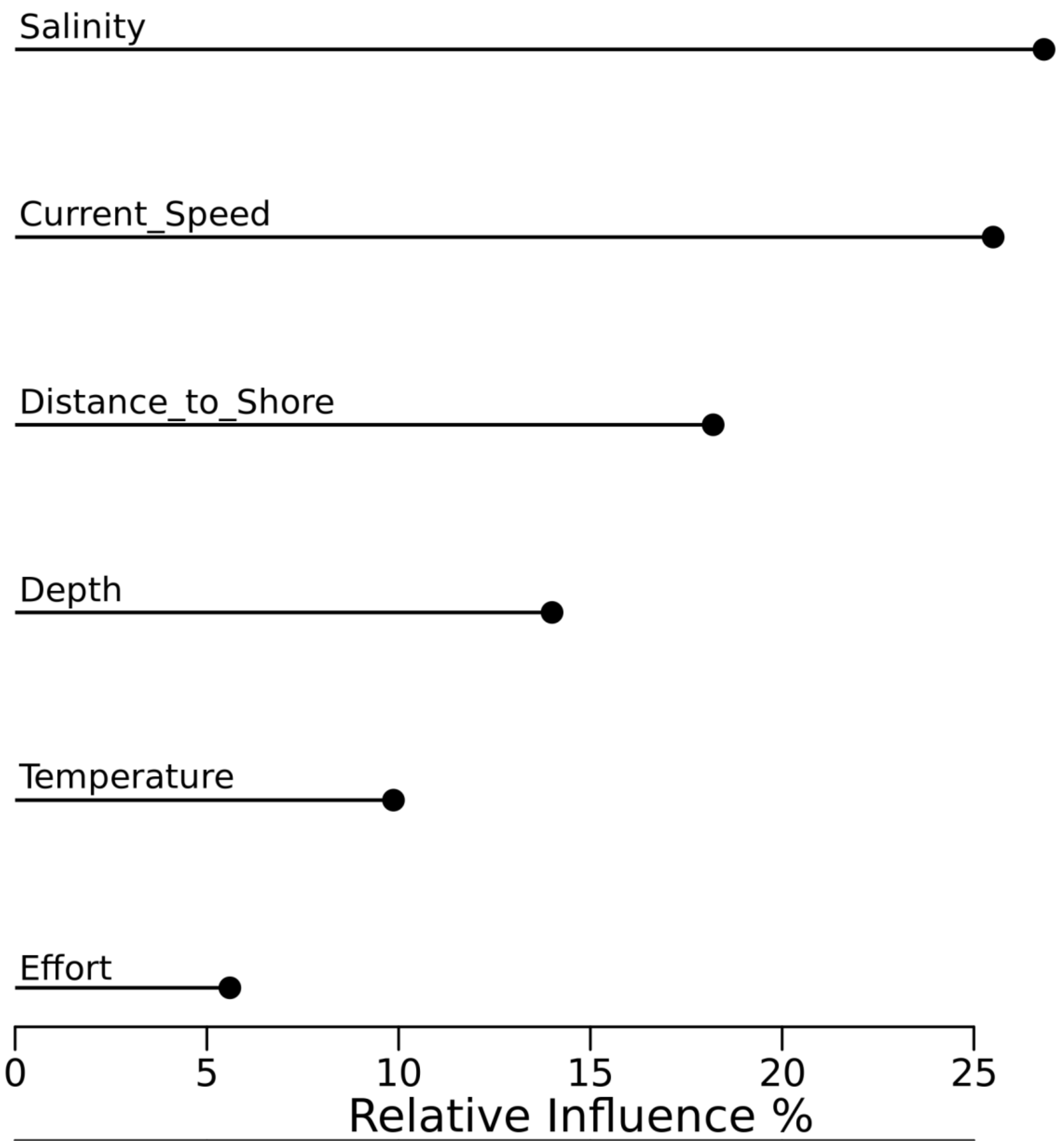

Fig 1. Bar plot of size of influence of variables, from *gbm.auto*

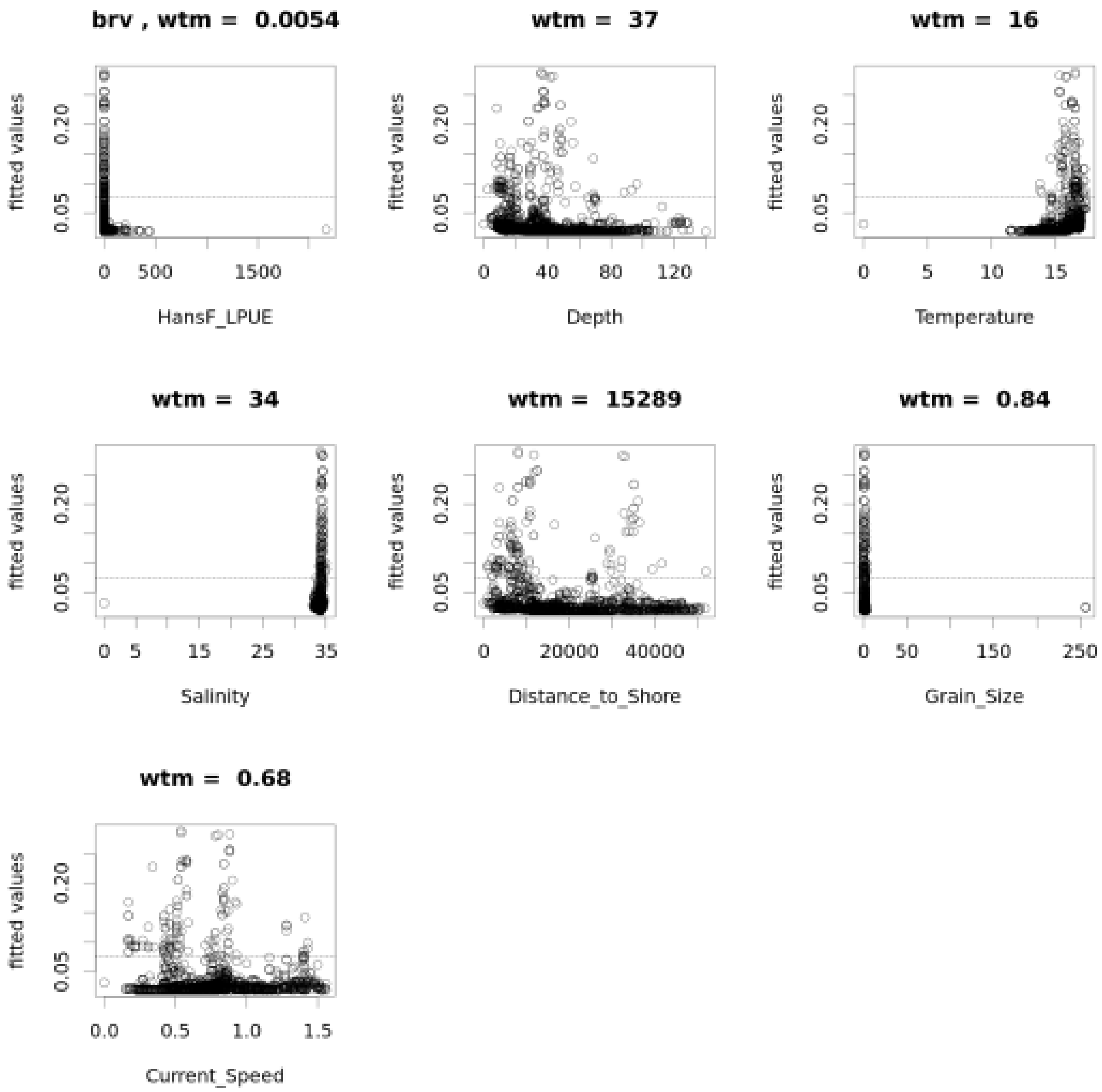

Fig 2. Dot plot matrix of spread of explanatory variables, from *gbm.auto*

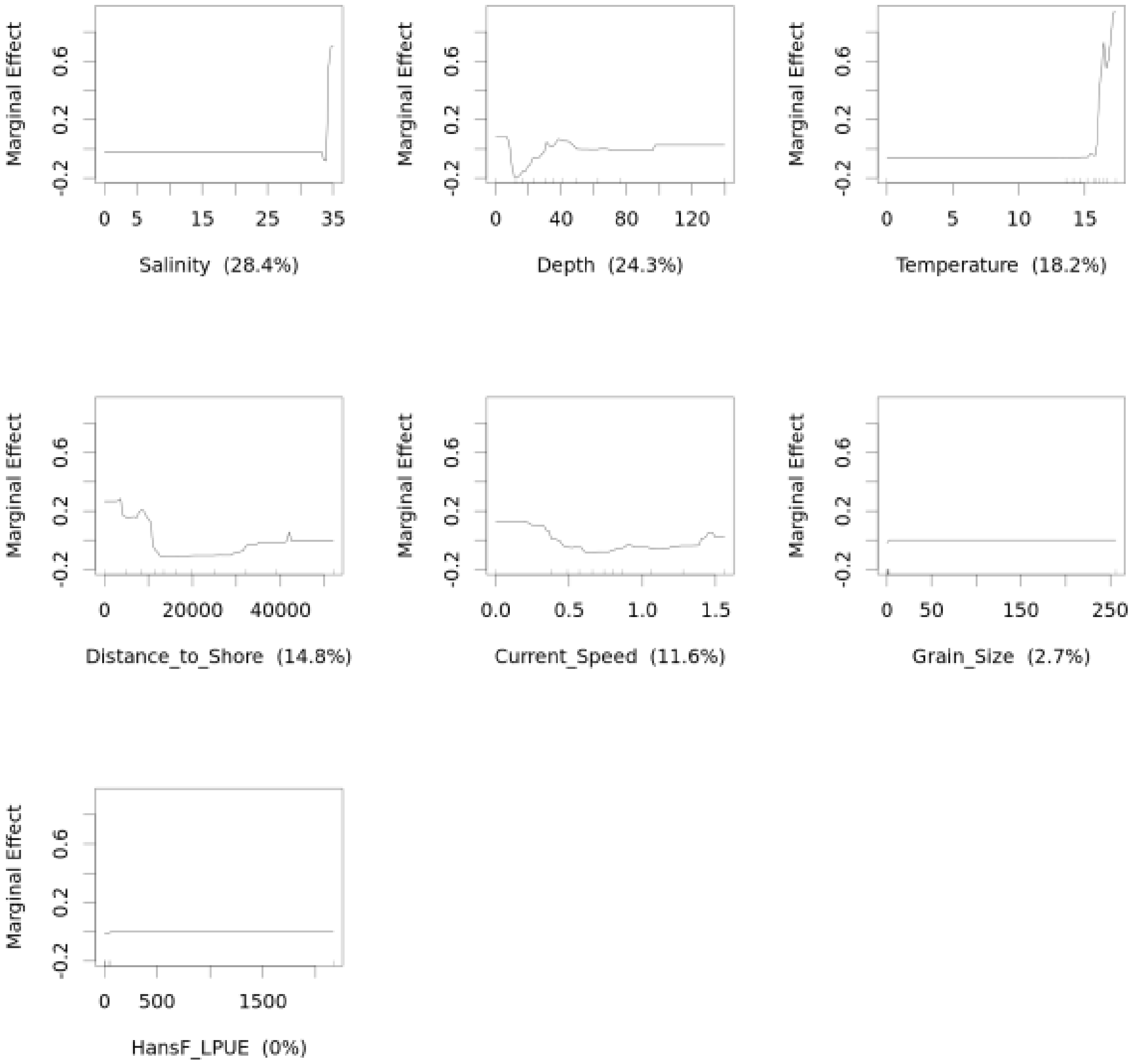

Fig 3. Line plot matrix of partial influence relationships of explanatory variables on the response variable, from *gbm.auto*

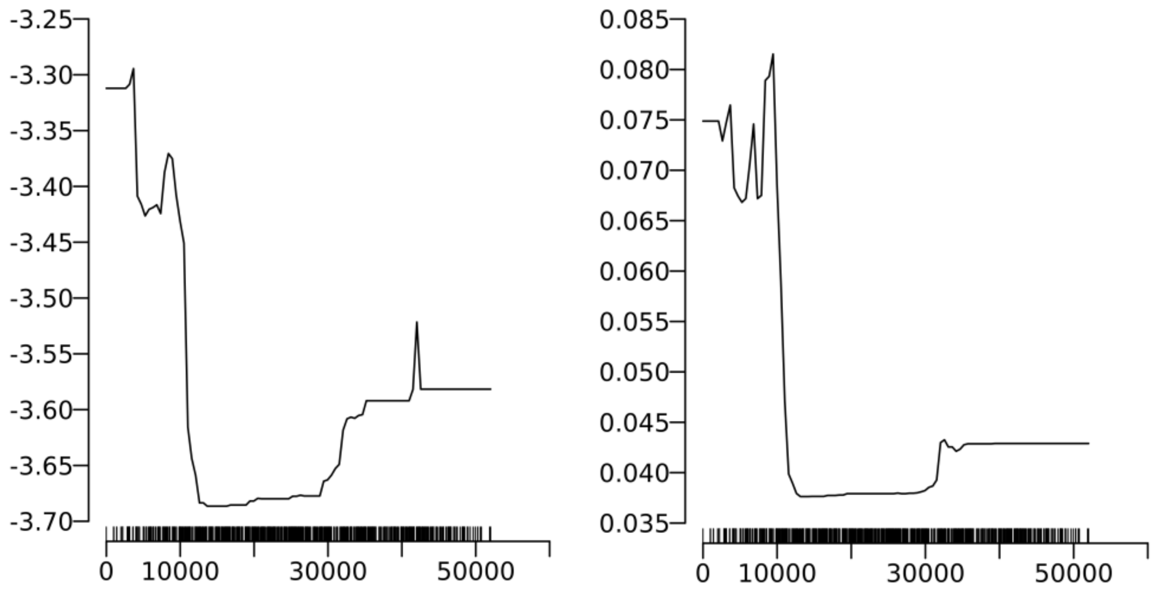

Fig 4. Partial influence relationships of binary (left) and Gaussian (right) explanatory variables (distance from shore) on the response variable, from *gbm.auto*

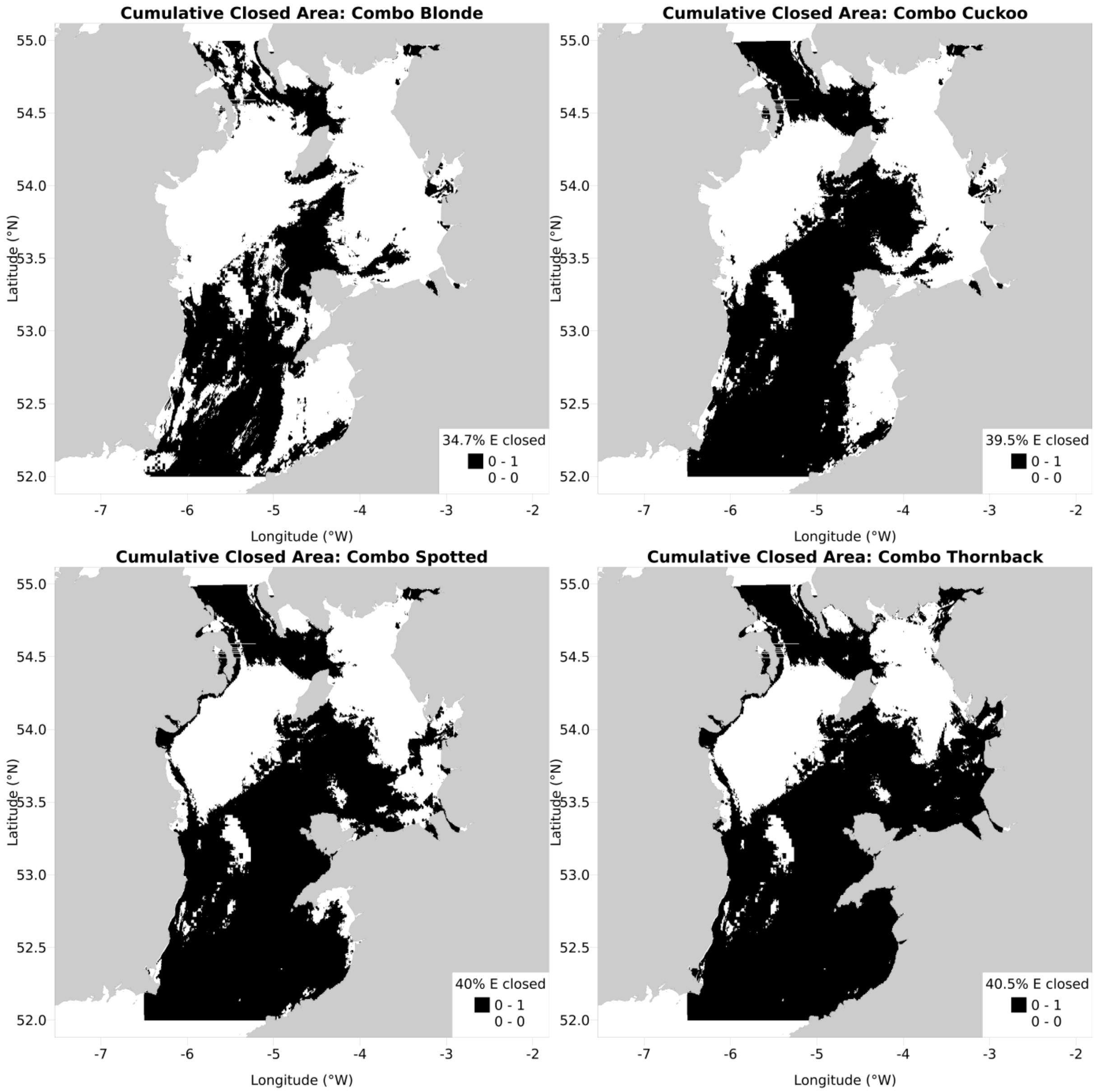

Fig 5. Growing closed-area-only maps, from *gbm.valuemap*

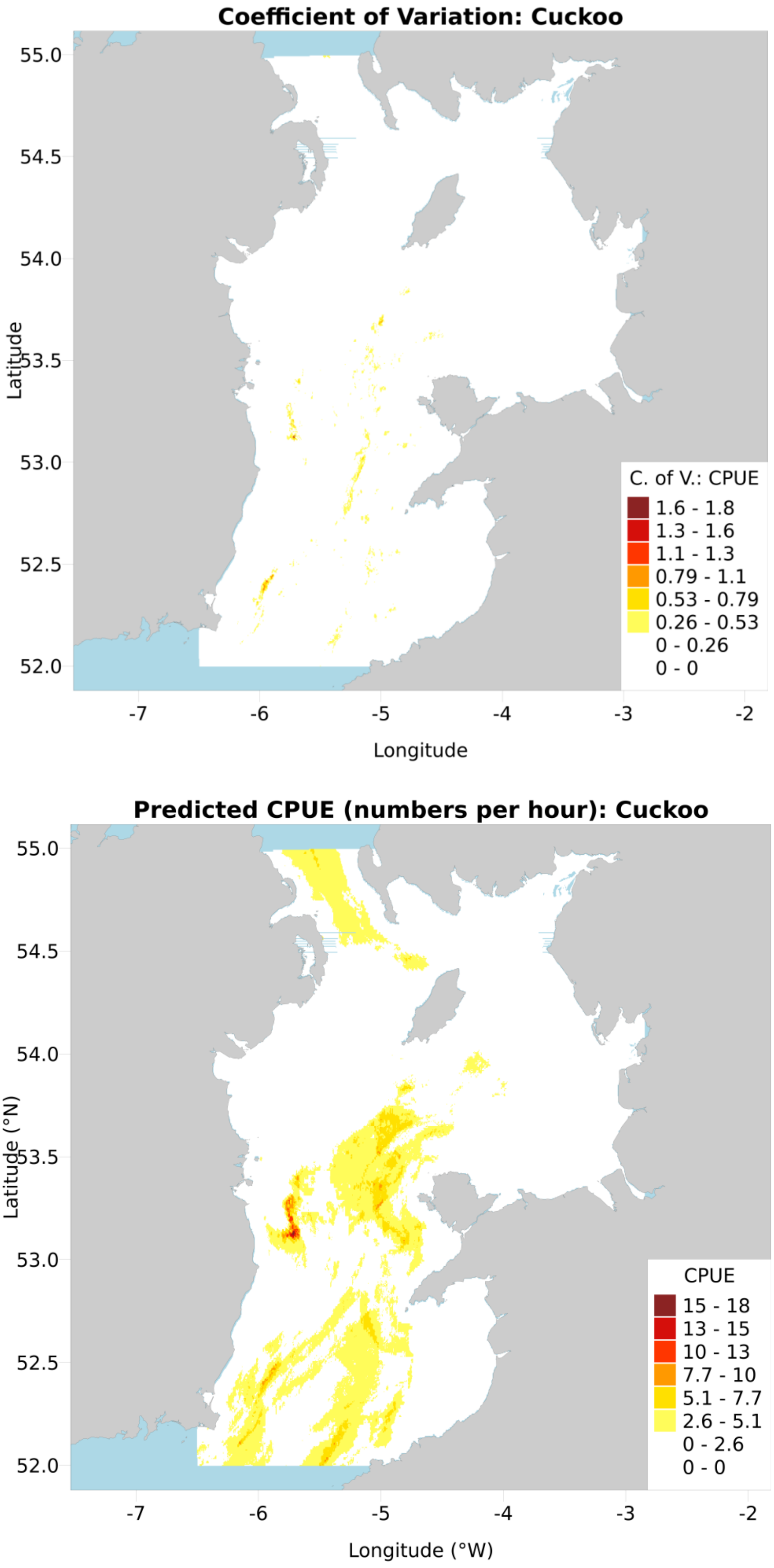

Fig 6 Coefficient of variation map of cuckoo ray CPUE, from *gbm.loop*; 6b: related predicted abundance map

In addition, the following data files and their contents are generated:
The report of all arguments: Explanatory Variables list, Response Variables list, Zero Inflated data test result, TC/LR/BF argument values for combination 1, BRT statistics for binary BRT for combination 1, BRT statistics for Gaussian BRT for combination 1, name for combination 1, values statistics and name for subsequent, best and simplified BRT combinations, best and dropped predictor variables for best binary and Gaussian BRTs, Relative Influence of binary and Gaussian predictors, biggest explanatory variable interactions for binary and Gaussian BRTs.
The RSB data file: Latitude, longitude, histogram difference score for each variable, modulus of the histogram difference score for each variable, binary unrepresentativeness, Gaussian unrepresentativeness, unrepresentativeness total.

The predicted abundance data including all explanatory data: Latitude, longitude, all explanatory variables, binary predicted abundance, Gaussian predicted abundance logged, Gaussian predicted abundance unlogged, final predicted abundance scores.

The Predicted abundance data with latitude and longitude only: Latitude, longitude, predicted abundance scores.

The *gbm.valuemap* report: Latitude, longitude, original response variables, scaled response variables, scaled and weighted response variables, individual species closures for all response variables separately (0 or 1), combined closed area for all response variables (0 or 1), *sum* closed area for all of n response variables (0 to n), cumulative closed area for all response variables (first, first and second, first and second and third, etc.). Closure statistics provided for all four sort methods.

The *gbm.loop* csvs: VarAll.csv with longitude, latitude, a column for the predicted abundance at each site for each run, and a final column of each site’s coefficient of variance, and; VarOnly.csv, just the latitudes, longitudes, and coefficients of variation.

5. References

1. Dedman S, Officer R, Brophy D, Clarke MW, Reid DG (2015) Modelling abundance hotspots for data-poor Irish Sea rays. Ecological Modelling 312: 77–90. doi:10.1016/j.ecolmodel.2015.05.010.

2. Dedman S, Officer R, Brophy D, Clarke MW, Reid DG (2016) Towards a flexible Decision Support Tool for MSY-based Marine Protected Area design for skates and rays. ICES Journal of Marine Science: fsw147. doi:doi:10.1093/icesjms/fsw147.

3. Parisien M-A, Moritz M (2009) Environmental controls on the distribution of wildfire at multiple spatial scales. Ecological Monographs 79: 127–154.

4. Elith J, Leathwick JR, Hastie T (2008) A working guide to boosted regression trees. Journal of Animal Ecology 77: 802–813.

5. Duan N (1983) Smearing estimate: a nonparametric retransformation method. Journal of the American Statistical Association 78: 605–610.
