# Supplementary material for: Gbm.auto: A software tool to simplify spatial modelling and Marine Protected Area planning
Source: PLoS One. 2017 Dec 7;12(12):e0188955. doi: 10.1371/journal.pone.0188955 (PMC5720763; doi:10.1371/journal.pone.0188955)
Supplement: S2 File — (PDF) [file pone.0188955.s002.pdf]

Gbm.auto: a software tool to simplify spatial modelling and Marine Protected Area planning

Simon Dedman[1,2*], Rick Officer[1], Maurice Clarke[2], David G. Reid[2], Deirdre Brophy[1]

[1] Marine and Freshwater Research Centre, Galway-Mayo Institute of Technology, Galway, Ireland; +1 415 944 7258

[2] Marine Institute, Rinville, Oranmore, Co. Galway, Ireland

* Corresponding author

Email: simondedman@gmail.com (SD)

Supplementary Material Appendix S2

Data sources and processing

All variables except the response variable need to be present in both the samples and grids datasets, and thus require processing to match the base resolution of each dataset. We downloaded CPUE data (in numbers per hour) for all rays caught in ICES area VIIa (Irish Sea) by standardised survey trawls (International Groundfish Survey and Bottom Trawl Survey) from 1993 to 2014 from the ICES Database of Trawl Surveys (ICES, 2015). To maximise the spatial coverage of the analysis, these data were averaged across all years. Cuckoo, thornback, blonde or spotted rays were present in 1645 of the 3341 half-hour trawls, the midpoints of which were used as the map locations for those data. This generated 1447 site locations, many of which were closely located because the surveys aim to re-sample the same sites. These are the 'samples' locations.

QGis mapping software (Thiede et al., 2013) was used to interpolate environmental point data to a Voronoi polygons surface (vector > geometry tools > Voronoi polygons), then to append their values to the highest resolution dataset (http://bit.ly/21AHBs6), which

was depth grids covering the whole Irish Sea (n = 391,568) – this is the basis for the ‘grids’ data. These environmental data were also appended to the samples points. Distance to shore was calculated using raster proximity analysis (http://bit.ly/1TuNRMd) and appended to both datasets. Surveyed fish predator CPUEs, also from the ICES Database of Trawl Surveys, were already at the samples locations, and were interpolated to the grids locations via Voronoi polygons in Qgis. Average annual ray and whelk LPUE, and scallop dredging effort, were all grid cell data to begin with, so they can be appended directly to the samples and grids points.

Table 1. Datasets used during modelling, and their sources. Ppm: parts per million. Mm: millimetres. $M.s^{-1}$: metres per second. M: metres. CPUE/LPUE: catch/landings per unit effort. Kg: kilogrammes. Hr: hour. KwH: Kilowatt-hour

Environmental Dataset	Spatial Resolution	Source
Depth	275x455m grids	EMODnet (European Marine Observation and Data Network)(EMODnet, 2014)
Average Monthly sea bottom temperatures 2010-2012 (°C), Average Monthly sea bottom salinities 2010-2012 (ppm), Maximum monthly 2 dimensional velocity ($m.s^{-1}$)	1185x1680m grids	Marine Institute, 2014 (http://www.marine.ie/Home/site-area/data-services/data-services)
Distance to shore (m)	275x455m grids	via European coastline layer (freely available)

Fishing & Predation Dataset	Spatial Resolution	Source
Surveyed ray CPUE (numbers per hour), 1990-2014	Point data (n=1447)	ICES DATRAS (ICES, 2015)
Surveyed fish predator CPUE (numbers per hour), 1990-2014	Point data	ICES DATRAS (ICES, 2015)
Average annual ray LPUE from demersal trawls (Kg^{-Hr}), 2006-2012	0.02° lat * 0.03° lon grids	Marine Institute, 2014
Average annual whelk LPUE (Kg^{-KwH}), 2009-2013	0.5° lat * 1° lon ICES rectangles	Marine Management Organisation, 2015
Average annual scallop dredging effort (KwH), 2006-2013/2014	0.5° lat * 1° lon ICES rectangles	Marine Management Organisation, and Marine Institute, 2015
Average annual scallop dredging effort (hours), 2006-2014	0.02° lat * 0.03° lon grids	Marine Institute, 2015

References

- EMODnet, 2014. EMODnet Biological Data Products [WWW Document]. URL http://bio.emodnet.eu (accessed 14–11.12).
- ICES, 2015. ICES Database of Trawl Surveys 1990 - 2014 [WWW Document]. URL http://datras.ices.dk (accessed 13–2.15).
- Thiede, R., Sutton, T., Düster, H., Sutton, M., 2013. Quantum GIS Training Manual, 1.0 ed.
