# Supplementary material for: Gbm.auto: A software tool to simplify spatial modelling and Marine Protected Area planning
Source: PLoS One. 2017 Dec 7;12(12):e0188955. doi: 10.1371/journal.pone.0188955 (PMC5720763; doi:10.1371/journal.pone.0188955)
Supplement: S3 File — (PDF) [file pone.0188955.s003.pdf]

Gbm.auto: a software tool to simplify spatial modelling and Marine Protected Area planning

Simon Dedman[1,2*], Rick Officer[1], Maurice Clarke[2], David G. Reid[2], Deirdre Brophy[1]

[1] Marine and Freshwater Research Centre, Galway-Mayo Institute of Technology, Galway, Ireland; +1 415 944 7258

[2] Marine Institute, Rinville, Oranmore, Co. Galway, Ireland

* Corresponding author

Email: simondedman@gmail.com (SD)

Supplementary Material Appendix S3

R functions and packages used

beepr: Bååth, R. 2015. Easily Play Notification Sounds on any Platform. R package version: 1.2. http://cran.r-project.org/package=beepr

calibration, *roc*, and *gbm.predict.grids*: from [1]'s appendix, built upon Friedman's work [2], bundled into *gbm.utils* by Dedman.

dismo: Hijmans, R.L., Phillips, S., Leathwick, J. and Elith, J. 2103. dismo: Functions for species distribution modelling, that is, predicting entire geographic distributions from occurrences at a number of sites. R package version: 0.9-3. http://cran.r-project.org/package=dismo

gbm: Ridgeway, G. 2013. gbm: Generalised Boosted Regression Models. R package version: 2.1. http://cran.r-project.org/package=gbm

mapplots: Gerritsen, H. 2014. mapplots: Data Visualisation on Maps. R package version 1.5. http://CRAN.R-project.org/package=mapplots

mgcv: Wood, S.N. 2011. Mgcv: Fast stable restricted maximum likelihood and marginal likelihood estimation of semiparametric generalized linear models. Journal of the Royal Statistical Society (B) 73(1):3-36. http://CRAN.R-project.org/package=mgcv

raster: Hijmans, R. J., van Etten, J., Cheng, J., Mattiuzzi, M., Sumner, M., Greenberg, J. A., Perpinan Lamiguueiro, O., Bevan, A., Racine, E. B., Shortridge, A., 2015. Raster: Geographic Data Analysis and Modeling. R package version 1.1-3. http://CRAN.R-project.org/package=raster

rgdal: Bivand, R., Keitt, T., Rowlingson, B., Pebesma, E., Sumner, M., Hijmans, R., Rouault, E., 2015. Rgdal: Bindings for the Geospatial Data Abstraction Library. R package version 1.1-3. http://CRAN.R-project.org/package=rgdal

vegan: Oksanen, J., Blanchet, F.G., Kindt, R., Legendre, P., Minchin, P.R., O'Hara, R.B., Simpson, G.L., Solymos, P., Stevens, M.H.H. and Wagner, H. 2013. vegan: Community Ecology Package. R package version 2.0-10. http://CRAN.R-project.org/package=vegan

R package functions *gbm.auto*, including *gbm.map*, *gbm.basemap*, *gbm.rsb*, *gbm.cons, gbm.valuemap, gbm.bfcheck, and gbm.loop* written by SD 2012-2017 and available at https://github.com/SimonDedman/gbm.auto. Version at time of publication is 1.1.3.

References

1. Elith J, Leathwick JR, Hastie T (2008) A working guide to boosted regression trees. Journal of Animal Ecology 77: 802–813.

2. Friedman JH (2001) Greedy function approximation: a gradient boosting machine. Annals of Statistics 29: 1189–1232.
